# Hematologic Ratios in Donkeys: Reference Intervals and Response to Experimentally Induced Endotoxemia

**DOI:** 10.3390/ani15152272

**Published:** 2025-08-04

**Authors:** Carmen Davias, Francisco J. Mendoza, Adelaida De Las Heras, Carlos Gonzalez-De-Cara, Antonio Buzon-Cuevas, Alejandro Perez-Ecija

**Affiliations:** 1 Department of Animal Medicine and Surgery, University of Cordoba, Campus Rabanales, 14014 Cordoba, Spain; carmendaviasm@gmail.com (C.D.); v02hesaa@uco.es (A.D.L.H.); carlosgonzalezdecara@gmail.com (C.G.-D.-C.); vetbuzon@gmail.com (A.B.-C.); alejandro.perez.ecija@uco.es (A.P.-E.); 2 Veterinary Teaching Hospital, University of Cordoba, Campus Rabanales, 14014 Cordoba, Spain

**Keywords:** Andalusian donkeys, endotoxemia, hematology, neutrophil to lymphocyte ratio, reference intervals, sepsis

## Abstract

Endotoxemia is commonly observed in donkeys, secondary to colic, pleuropneumonia, or diarrhea among other disorders. Hematologic ratios are new biomarkers widely used in the diagnosis and prognosis of multiple conditions in human medicine, including sepsis. While the utility of these ratios has been proved in septic foals, no data are available on donkeys. Moreover, reference intervals (RIs) have not been studied in this species. In this study, RIs of the most commonly reported hematologic ratios were determined in 73 healthy adult donkeys. In addition, variations in these ratios in response to LPS infusion were also evaluated in six healthy adult donkeys. Most of the ratios evaluated showed significant variations after induced endotoxemia, with most of them showing values outside of the established RIs. Similarly to septic foals, the neutrophil to lymphocyte ratio was significantly reduced after LPS infusion. No significant changes were observed in the red cell distribution width to platelet ratio, contrary to reports on septic foals. Previously reported cut-off values for both of these ratios should not be extrapolated to donkeys. Future studies evaluating these ratios in natural endotoxemia or other diseases in donkeys, as well as establishing species-specific cut-off values, are necessary.

## Simple Summary

Donkeys commonly suffer from endotoxemia as a complication of other diseases such as colic, pleuropneumonia, or diarrhea. Although new hematologic ratios are used in human medicine and foals to diagnose and determine the outcome in sepsis, no data are available in donkeys. Reference intervals for these ratios were established in adult healthy donkeys. LPS infusion caused significant variations in most of the ratios studied, with changes in the neutrophil to lymphocyte ratio being similar to the ones reported in septic foals. However, no changes in the red cell distribution width to platelet ratio were observed, contrary to septic foals. Moreover, reported cut-off values in foals should not be used in donkeys. In conclusion, these ratios are informative for donkeys and could be useful in the diagnosis of endotoxemia. More studies evaluating the changes in these ratios in different diseases and specific cut-off values in this species are necessary.

## 1. Introduction

Donkey medicine is a growing area of interest due to the increasing use of this species as pets, in assisted therapy, alimentary by-products production, and novel sport activities, as well as its traditional role in transport and agriculture in developing countries [1]. Hematology is one of the most important and commonly used diagnostic techniques in donkey medicine, useful for health screening, disease diagnosis, prognosis, and the monitoring of response to treatment. Reference intervals (RIs) for hematological parameters in donkeys have been reported using different automatic analyzers and specifically in certain endangered breeds [2,3,4]. These reports have shown marked differences compared to horses and other equids, which advise against the extrapolation of data between species and underlines the necessity of species-specific studies.

Similarly to horses, endotoxemia is a common disorder in donkeys, mostly secondary to colic, diarrhea, and pleuropneumonia among other disturbances [5]. This condition is linked to high mortality and serious complications such as hyperlipemia, laminitis, disseminated intravascular coagulation, and multiorgan dysfunction [6]. Information on the pathogenesis, diagnosis, treatment, and outcome of endotoxemia is scarce in donkeys [7,8,9]. Previous reports from our research group have characterized the effects of experimentally induced endotoxemia on basic hematology, hemodynamic parameters, acute-phase protein concentrations, coagulation, and inflammatory cytokines [6,9,10,11]. Because clinical signs of sepsis are nonspecific, early diagnosis is challenging to veterinarians. However, an early diagnosis is crucial in order to establish an oriented treatment and to increase the probability of a successful outcome [12].

Hematologic ratios are emerging biomarkers with utility in the diagnosis and prognosis of a wide variety of diseases in human medicine, such as neurologic conditions, myocardial pathologies, or cancer [13,14,15]. These ratios are also widely used in human sepsis as reliable and early indicators of bacteriemia or septic shock and are also linked to the outcome of the patient [16,17,18]. Compared to other sepsis biomarkers (cytokines, acute-phase proteins, etc.), hematologic ratios are easy and fast to determine and do not require specialized personal or advanced techniques. These ratios are also superior to white blood cells counts and the absolute number of leukocyte subpopulations for sepsis diagnosis [16]. Although there is scarce information about these ratios in equids, recent studies have shown their utility in equine piroplasmosis and described their prognostic value in septic foals [12,19,20,21], with some of them being recognized as potential clinical pathology markers for scoring systems in septic foals [22]. To the authors’ knowledge, there is no current information on the utility of these ratios in donkey endotoxemia. Moreover, RIs for these ratios have not been published on this species.

The hypothesis of this work was that acute endotoxemia would induce changes in hematologic ratios in donkeys. Therefore, the objectives of this study were: (a) to characterize the RIs of hematologic ratios in healthy donkeys, and (b) to determine the changes in these hematologic ratios in response to experimentally induced endotoxemia in healthy donkeys.

## 2. Materials and Methods

### 2.1. Animals Selection and Blood Collection for RI Calculation in Healthy Donkeys

Data were analyzed retrospectively from a database of blood samples collected for a health check programme over a period of 2 years performed in several semi-extensive farms in Southern Spain (Andalusia). Only clinically healthy donkeys were included in this study. Animals were considered healthy based on both a normal clinical history and physical examination: heart and respiratory rates, body temperature, mucous membrane colour, capillary refill time, intestinal motility in the four quadrants, and digital pulse. All donkeys were under a deworming programme (either based on a regular blanket treatment or on a fecal egg count) and had not received any treatment for at least one month prior to sampling.

A total of 73 donkeys (61 mares, 8 stallions and 4 geldings) fulfilled these criteria and were used to calculate the RIs. These donkeys had a mean age of 8.6 ± 3.5 years (range of 4 to 20 years), with most animals being of the Andalusian breed (65 Andalusian and 8 crossbred).

All blood samples were collected in the morning in each farm by jugular venipuncture using an 18-gauge, 1.5-inch needle and a 5 mL syringe (Terumo, Madrid, Spain). K_3_-EDTA tubes (Becton Dickinson, Plymouth, UK) were used. Blood samples were chilled on ice. Once back in the laboratory, samples were mixed on a rocker (Auxilab S.L., Navarra, Spain) for 30 min prior to measurement. Time from blood collection to measurement was less than 12 h.

### 2.2. Endotoxemia Induction

Six healthy adult (7.6 ± 0.8 years old) Andalusian, non-pregnant jennies (348.3 ± 38.9 kg) housed in the facilities of the Veterinary Teaching Hospital of the University of Cordoba were used for the second objective. Animals were considered healthy based on a normal clinical history, physical examination (as previously detailed), and a complete blood work profile (complete blood count, total protein, albumin, creatinine, urea, aspartate transaminase -AST-, gamma-glutamyl transferase -γGT-, and total bilirubin concentrations). All the donkeys were dewormed prior to transport to the hospital and had not received any treatment for at least 2 months before the experiment. These animals were part of a larger research project about donkey endotoxemia.

Endotoxemia was induced following protocols previously described both in donkeys and horses [9,23]. Two polyurethane catheters (Milacath, Mila International Inc., Hebron, KY, USA) were placed aseptically in both jugular veins. A volumetric pump (Infusomat, Braun VetCare, Barcelona, Spain) was used to administer 20 ng/Kg of LPS (*Escherichia coli* O55:B5, Sigma-Aldrich Quimica, Madrid, Spain) diluted in 500 mL of saline solution over 30 min in the left jugular vein (from −30 to 0 min). Blood samples were collected from the right jugular vein catheter into K_3_-EDTA tubes (Becton Dickinson, Plymouth, UK) at the following times: −30 (baseline), 0 (end of LPS infusion), and 30, 60, 90, 120, 150, 180, 240, and 360 min post-LPS infusion (PLI).

This study received approval from the Welfare Committee of Animal Research from the University of Cordoba (2015PI/05) and the regional Government (19-03-15-212, Consejeria de Salud y Familias, Junta de Andalucia). Animals were handled according to national guidelines for research animals.

### 2.3. Hematologic Parameters Determinations and Ratios Calculation

An automated hematology analyzer (Lasercyte, Idexx Laboratories SL, Hoofddorp, the Netherlands), previously validated in donkeys [4], was used to determine the following parameters: white blood cell count (WBC), neutrophil concentration (NEU), lymphocyte concentration (LYM), monocyte concentration (MONO), eosinophil concentration (EOS), red cell distribution width (RDW), and platelet counts (PLT). An internal quality assessment was performed weekly using the manufacturer’s quality control material (LaserCyte CBC5R test kit, Idexx Laboratories Incorporation, Hoofddorp, the Netherlands).

The following hematologic ratios were calculated based on the results from the analyzer: the neutrophil to lymphocyte ratio (NLR), neutrophil to monocyte ratio (NMR), lymphocyte to monocyte ratio (LMR), monocyte to lymphocyte ratio (MLR), eosinophil to lymphocyte ratio (ELR), platelet to WBC ratio (PWR), platelet to neutrophil ratio (PNR), platelet to lymphocyte ratio (PLR), platelet to monocyte ratio (PMR), and the red cell distribution width to platelet ratio (RDWPLT) [13,14,15,16,17,18,19,20,21,22].

### 2.4. Statistical Analysis

Normality was assessed by a Kolmogorov–Smirnov test. Results were expressed as the mean ± standard error of the mean (SEM) or median and interquartile range (IQR = 75th–25th percentiles) as appropriate. The median and percentiles were calculated using Tukey’s Hinges test.

Following recommendations from the American Society of Veterinary Clinical Pathology, reference intervals were obtained with a robust or nonparametric method as appropriate, using a dedicated software (Reference Value Advisor v. 2.1. freeware. Available at: http://www.biostat.envt.fr/reference-value-advisor/ (Accessed on 18 June 2025)), and are presented as two-sided 90% confidence intervals [24]. This software tests the normality of the introduced data, identifies outliers, and calculates reference limits with their confidence intervals using a nonparametric method when *n* ≥ 40 and by parametric and robust methods from native and Box–Cox transformed values [25].

Differences among time-points during induced endotoxemia were studied using an ANOVA of repetitive measures, followed by a Bonferroni post hoc analysis or a Friedman test, followed by a Dunn’s post hoc analysis, depending on the distribution.

Statistical analyses were performed using a statistical software (GraphPad Prism 9, San Diego, CA, USA), and values with *p* < 0.05 were considered significant.

## 3. Results

### 3.1. RIs for Hematologic Ratios in Healthy Donkeys

Reference intervals for every hematologic ratio are compiled in Table 1. Frequency distributions of these variables are compiled in Supplementary Figures S1 and S2. Hematologic and platelets-related parameters were partially published in a previous comparative study [4].

### 3.2. Changes in Hematologic Ratios in Response to Experimentally Induced Endotoxemia

All donkeys developed typical features of a systemic inflammatory response syndrome (SIRS) such as tachycardia, fever, and an increase in plasma TNFα and IL-1β concentrations from 30 min PLI (data previously published) [9].

**Figure 1:**
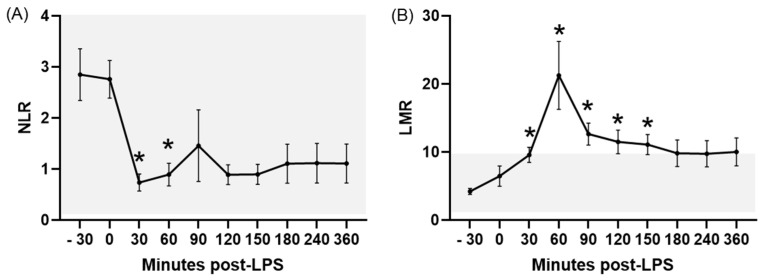
NLR (**A**) and LMR (**B**) in donkeys after LPS infusion. Bar represents the standard error of the mean. The grey area represents the reference ranges for healthy donkeys. * *p* < 0.05 vs. −30 min (baseline). LMR, lymphocyte to monocyte ratio; NLR, neutrophil to lymphocyte ratio.

The NLR showed a rapid response to LPS infusion, with statistically significant decreases at 30 and 60 min PLI compared to the baseline (*p* ≤ 0.04). Values remained low until the end of the experiment (Figure 1A). The NLR was within the RIs for healthy donkeys (established in the first objective) during the entire experiment.

No statistically significant variations were observed in the NMR, although a rapid decrease was observed at 30 min PLI (Supplementary Figure S3A). Values remained within the RIs established previously.

LPS infusion caused a significant (*p* < 0.05) increase in the LMR between 30 and 150 min PLI (Figure 1B), with values above the RIs for healthy donkeys between 60 and 150 min PLI.

**Figure 2:**
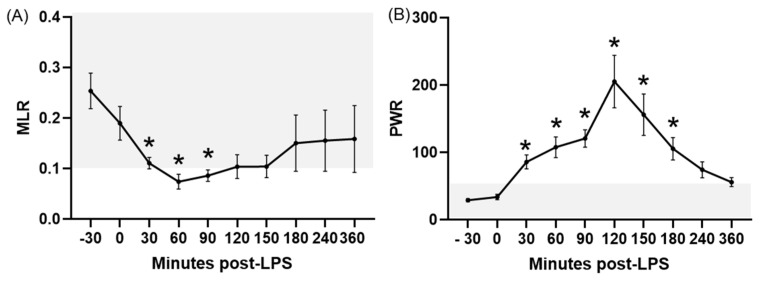
MLR (**A**) and PWR (**B**) in donkeys after LPS infusion. Bar represents the standard error of the mean. The grey area represents the reference ranges for healthy donkeys. * *p* < 0.05 vs. −30 min (baseline). MLR, monocyte to lymphocyte ratio; PWR, platelet to white blood cell ratio.

The MLR significantly (*p* ≤ 0.02) decreased at 30, 60 and 90 min PLI, with values below the reference intervals for healthy donkeys at 60 and 90 min PLI (Figure 2A). No return to baseline was observed at the end of the experiment.

Although an increase at 90 min PLI was observed in the ELR (Supplementary Figure S3B), no statistical differences were noted at any time.

**Figure 3:**
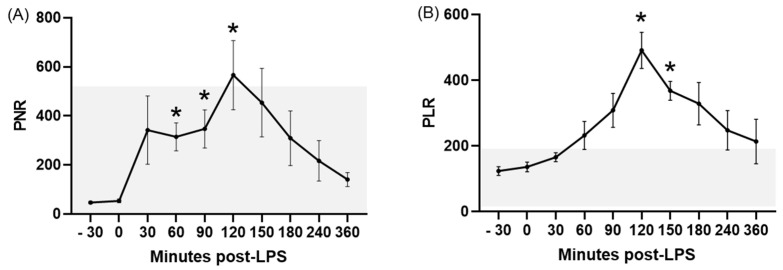
PNR (**A**) and PLR (**B**) in donkeys after LPS infusion. Bar represents the standard error of the mean. The grey area represents the reference ranges for healthy donkeys. * *p* < 0.05 vs. −30 min (baseline). PLR, platelet to lymphocyte ratio; PNR, platelet to neutrophil ratio.

**Figure 4:**
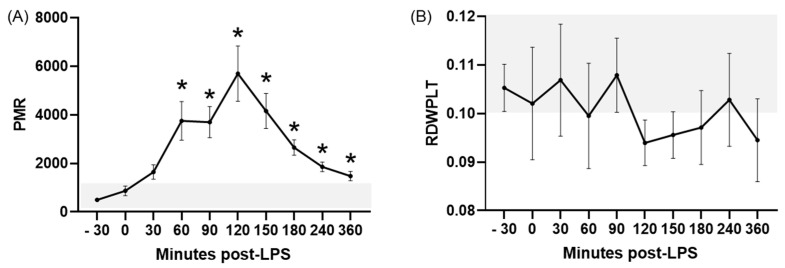
PMR (**A**) and RDWPLT (**B**) in donkeys after LPS infusion. Bar represents the standard error of the mean. The grey area represents the reference ranges for healthy donkeys. * *p* < 0.05 vs. −30 min (baseline). PMR, platelet to monocyte ratio; RDWPLT, red cell distribution width to platelet ratio.

Concerning platelet-related ratios, LPS infusion caused significant increases in all of them except for the RDWPLT. The PWR showed a significant (*p* ≤ 0.03) increase compared to the baseline between 30 and 180 min PLI (Figure 2B), whereas this change was limited to the periods ranging between 60 and 120 min PLI for the PNR (*p* ≤ 0.02; Figure 3A), 120–150 min PLI for the PLR (*p* ≤ 0.004; Figure 3B), and between 60 and 360 min PLI for the PMR (*p* ≤ 0.03; Figure 4A). In most of these ratios, time-points after LPS infusion showed values above the RIs for healthy donkeys, except for the PNR. No significant differences were noted for the RDWPLT, although a trend to values below the RIs was observed (Figure 4B).

## 4. Discussion

Hematologic ratios are emerging biomarkers in human medicine, which have been demonstrated to be helpful for the diagnosis, prognosis, and outcome in multiple conditions [13,14,15,16,17,18]. Scarce information is available about these ratios in veterinary medicine, with few reports on small animals [26,27] and even fewer on horses, mostly focused on neonates [12,20,21,22]. These veterinary reports usually discuss the use of these ratios in infectious diseases, but RIs in healthy animals are not frequently determined. In equids, RIs have only been published for NLR and RDWPLT in foals [12,20], although not completely following the recommendations from the American Society of Veterinary Clinical Pathology. To our knowledge, this report is the first one describing the RIs for the most common hematologic ratios in healthy donkeys. These results are fundamental for future studies evaluating variations in these parameters in different diseases in this species.

Acute experimentally induced endotoxemia caused significant changes in most of the hematologic ratios studied, characterized by a diminished NLR and MLR and elevated LMR, PWR, PNR, PLR, and PMR. In the majority of these ratios, variations induced by LPS corresponded to values outside of the RIs established for healthy donkeys in the first objective of this study. To the authors’ knowledge, this is also the first report of these ratios in endotoxemic donkeys, showing their potential utility in the early diagnosis of this disorder in this species.

The NLR is commonly used in human medicine as a biomarker to predict disease severity, outcome, and survival in bacteremia and sepsis [28,29]. This ratio responds rapidly to infections and is a better predictor of bacteremia than acute-phase proteins [17]. In equine foals, the NLR has proved to be different between healthy, sick non-septic hospitalized foals, and septic foals, with the last group showing statistically lower values [20]. Moreover, foals with a low NLR have a worse prognosis, as it is linked to higher odds of non-survival [20,21]. Our results in donkeys concur with these previous reports, demonstrating a rapid and significant decrease in the NLR in response to LPS infusion. This response is opposite to the one reported in humans, dogs, and cats, where a higher NLR is seen in response to infections and sepsis, and high NLR values are correlated to a poor prognosis and worse disease progression [26,27,30]. This idiosyncrasy in equids could be explained by the tendency of donkeys and horses to present neutropenia without significant variations in lymphocytes in response to endotoxemia instead of neutrophilia and concurrent lymphopenia, as seen in other mammals [20]. The NLR has also been studied in equine piroplasmosis, with significantly lower values observed in horses serologically positive to *Theileria equi* and *Babesia caballi* compared to the control group [19]. Reference intervals for the NLR in healthy donkeys were similar to those reported in humans and dogs [26,31]. Although there are no reports about the RIs of this ratio in adult horses, intervals in donkeys were narrower than those previously reported in foals [20].

In a recent study, a cut-off value of NLR < 3.06 was established to identify sick foals at risk of sepsis at admission [20]. Since our healthy donkeys already showed an NLR < 3.06 at the beginning of the experiment, prior to LPS administration, this cut-off value could not be extrapolated to this species. This finding emphasizes the need for species-specific studies and cut-off values.

One previous study demonstrated a negative correlation between NLR values and the gene expression of the pro-inflammatory cytokine IL-1β in septic foals [32]. Similarly, the decrease in NLR in our donkeys was in consonance with the elevation, both in plasma and mRNA concentrations, of the pro-inflammatory cytokines TNF-α and IL-1β in endotoxemic donkeys [9].

In human medicine, low LMR values are predictive of SIRS and a poor prognosis in septic patients [33,34]. This ratio also correlates with a poor prognosis in patients with cardiac damage, colorectal cancer, and influenza infection [14,15,35]. In our study, acute induced endotoxemia caused a significant LMR increase. This finding could be explained by differences in leukocyte dynamic relationships in response to neuroendocrine stress and immune–inflammatory imbalances between equids and humans, as previously mentioned for the NLR [20]. The LMR has not been evaluated in septic horses previously, although one previous study reported significantly higher LMR values in horses seropositive to *T. equi* [19].

High MLR values have been linked to mortality in human patients undergoing cardiac surgery [14]. In Arabian horses, high MLR values were also observed in response to endurance exercises, which were linked to decreased lymphocyte concentrations secondary to the inflammatory response or stress induced by the strenuous exercise [36]. Endotoxemic donkeys showed a significant decrease in MLR. This finding is similar to the one described in seropositive horses to *T. equi* [19]. These discrepancies in ratios among studies could be explained by species-specific idiosyncrasies and by differences in the pattern of inflammatory responses depending on the etiological agent or insult, as previously proposed [12].

Concerning the rest of the leukocyte-related hematologic ratios (NMR and ELR), no information is available on septic or endotoxemic horses in order to compare with our results.

Elevated RDWPLT values are correlated to a sepsis diagnosis, worse prognosis, and non-survival both in adult and neonatal human patients and foals [12,37,38]. In our study, we did not find significant variations in RDWPLT in response to induced endotoxemia in donkeys. Donkeys displayed wider and higher RIs values compared to those previously described in humans and foals [12,39].

A cut-off value of 0.09 for RDWPLT accurately predicts sepsis in foals [12]. Since RDWPLT values were higher in our healthy donkeys prior to LPS infusion, this cut-off cannot be used in donkeys and extrapolation to this species could lead to false diagnoses and unnecessary treatments.

In regard to the rest of the platelet-related ratios, a high PLR is described in human patients suffering from sepsis or SIRS [34,40]. Moreover, the PLR is also an accurate indicator of a poor prognosis in cardiovascular patients [14]. Recently, differences in the PLR value were reported between healthy horses and those with inflammatory conditions (according to acute-phase proteins) [41]. Our results in donkeys are similar to those reported in septic humans and horses with inflammation, thus demonstrating that platelets also play a substantial role in early inflammation and immunity in this species [42].

Neither PWR, PNR, nor PMR are deemed useful in the diagnosis or prognosis of human sepsis [13,43]. In contrast, they are considered valuable tools for other disturbances. For example, the PWR is strongly associated with mortality in humans with COVID-19, stroke, acute heart failure, and myocardial infarction; the PNR is related to the outcome in patients with acute ischemic stroke; and the PMR serves as a predictor of mortality in Hepatitis B Virus-associated cirrhosis [13,44,45]. Donkeys responded to LPS infusion with significant elevations in all of these ratios, with values above the RIs established for the species and variations maintained until the end of the experiment. As previously seen in other ratios, the direction of these changes was the opposite in our donkeys to those previously described in human patients, which could point to intrinsic differences between species in their response to sepsis and inflammation.

Hematologic ratios are regarded as early markers of inflammation and sepsis in human medicine, with their changes even preceding alterations in acute-phase proteins, such as serum amyloid A (SAA), presepsin, or C-reactive protein [16,17]. This appears to also be true in donkeys, since changes in most of the ratios were more rapid than the reported variations in SAA or haptoglobin (two hours post-infusion) [10]. This finding highlights that the hematologic ratios could be a reliable, fast, and cost-effective tool in the early diagnosis of sepsis in donkeys, more precise than other biomarkers commonly used by equine practitioners such as fibrinogen or SAA. However, it would be worth mentioning that most authors in human medicine recommend using these ratios in combination with other biomarkers, such as cytokines, acute-phase proteins, or sepsis scores [17].

The main limitation of this study is the lack of comparable studies concerning these ratios in veterinary medicine. The reports mentioned in foals were retrospective studies performed in hospitals and evaluated foals suffering from sepsis at admission. Thus, these data correspond to animals with heterogenous disease progressions and likely a systemic organ impairment, compared to our experimental design focused on acute short-term and reversible experimentally induced endotoxemia. In this sense, a more prolonged sampling post-LPS or a more severe endotoxemia degree could have found changes in these ratios more closely resembling those encountered by clinicians. Variations due to age (foals vs. adults) and etiological origin of the sepsis in clinical cases could have also influenced these ratios, limiting comparisons among studies. Concerning the variable age, it would have been ideal to compare donkey foal results to available foal data and/or our results to adult horse data (not published) before performing an interspecies comparison. Other shortcomings of this study are the small sample size, the long period used for the study of RIs (since circannual and circadian rhythmicity could be present in some hematological parameters), and the absence of blood smears (which could have been of value in order to confirm the results of the automatic analyzer). We initially calculated the RIs on a larger population (*n* = 90) including donkeys with ages between 1 and 4 years old. However, since some authors have noted hematological differences in this range of age with older donkeys [46,47], we only present data from mature animals. Nonetheless, RIs for these ratios were similar when those animals were included (Supplementary Table S1).

## 5. Conclusions

Most of the hematologic ratios studied showed significant variations in response to acute induced endotoxemia in donkeys. While donkeys with endotoxemia presented a significant decrease in NLR, proposed cut-off values for septic equine foals should not be extrapolated to donkeys in order to avoid a misdiagnosis. Contrary to septic equine foals, no variations were observed in RDWPLT in our donkeys. Future studies in naturally septic donkeys could further confirm our results and establish species-specific cut-off values for these ratios.

## Figures and Tables

**Table 1:** Reference intervals for hematologic ratios in healthy donkeys (*n* = 73).

Ratio	LRL of RI	URL of RI	CI 90% of LRL	CI 90% of URL
NLR (Neutrophil to Lymphocyte)	0.1	4.0	0.1–0.2	2.8–5.2
NMR (Neutrophil to Monocyte)	0.2	18.3	0.2–0.4	13.3–21.4
LMR (Lymphocyte to Monocyte)	1.2	9.9	1.1–2.2	9.1–10.0
MLR (Monocyte to Lymphocyte)	0.1	0.9	0.1–0.1	0.5–0.9
ELR (Eosinophil to Lymphocyte)	0.0	0.6	0.0–0.0	0.4–0.8
PWR (Platelet to WBC)	3.1	49.4	3.1–5.5	39.8–51.0
PNR (Platelet to Neutrophil)	6.6	565.0	6.0–11.0	281.3–714.3
PLR (Platelet to Lymphocyte)	8.2	194.4	7.7–12.3	151.3–247.4
PMR (Platelet to Monocyte)	41.3	924.9	20.6–61.5	667.8–1112.9
RDWPLT (RDW to Platelet)	0.1	0.8	0.1–0.1	0.6–0.9

CI, confidence interval; LRL, lower reference limit; RI, reference interval; URL, upper reference limit; and WBC, white blood cell count.

## Data Availability

The data presented in this study are available upon request to the corresponding author.

## Supplementary Materials

The following supporting information can be downloaded at: https://www.mdpi.com/article/10.3390/ani15152272/s1, Figure S1: Frequency distributions and reference intervals for NLR, NMR, LMR, MLR, and ELR in healthy adult donkeys; Figure S2: Frequency distributions and reference intervals for PWR, PNR, PLR, PMR, and RDWPLT in healthy adult donkeys; Figure S3: NMR (A) and ELR (B) in donkeys after LPS infusion. Bar represents the standard error of the mean. The grey area represents the reference ranges for healthy donkeys. * *p* < 0.05 vs. −30 min (baseline). NMR, neutrophil to monocyte ratio; ELR, eosinophil to lymphocyte ratio; and Table S1: reference intervals for hematologic ratios in healthy donkeys, included animals with ages between 1 and 4 years old (*n* = 90).
