# Supplementary material for: Hematologic Ratios in Donkeys: Reference Intervals and Response to Experimentally Induced Endotoxemia
Source: Animals (Basel). 2025 Aug 4;15(15):2272. doi: 10.3390/ani15152272 (PMC12345425; doi:10.3390/ani15152272)
Supplement: Supplementary file 1 [file animals-15-02272-s001.zip › animals-3758677-supplementary.pdf]

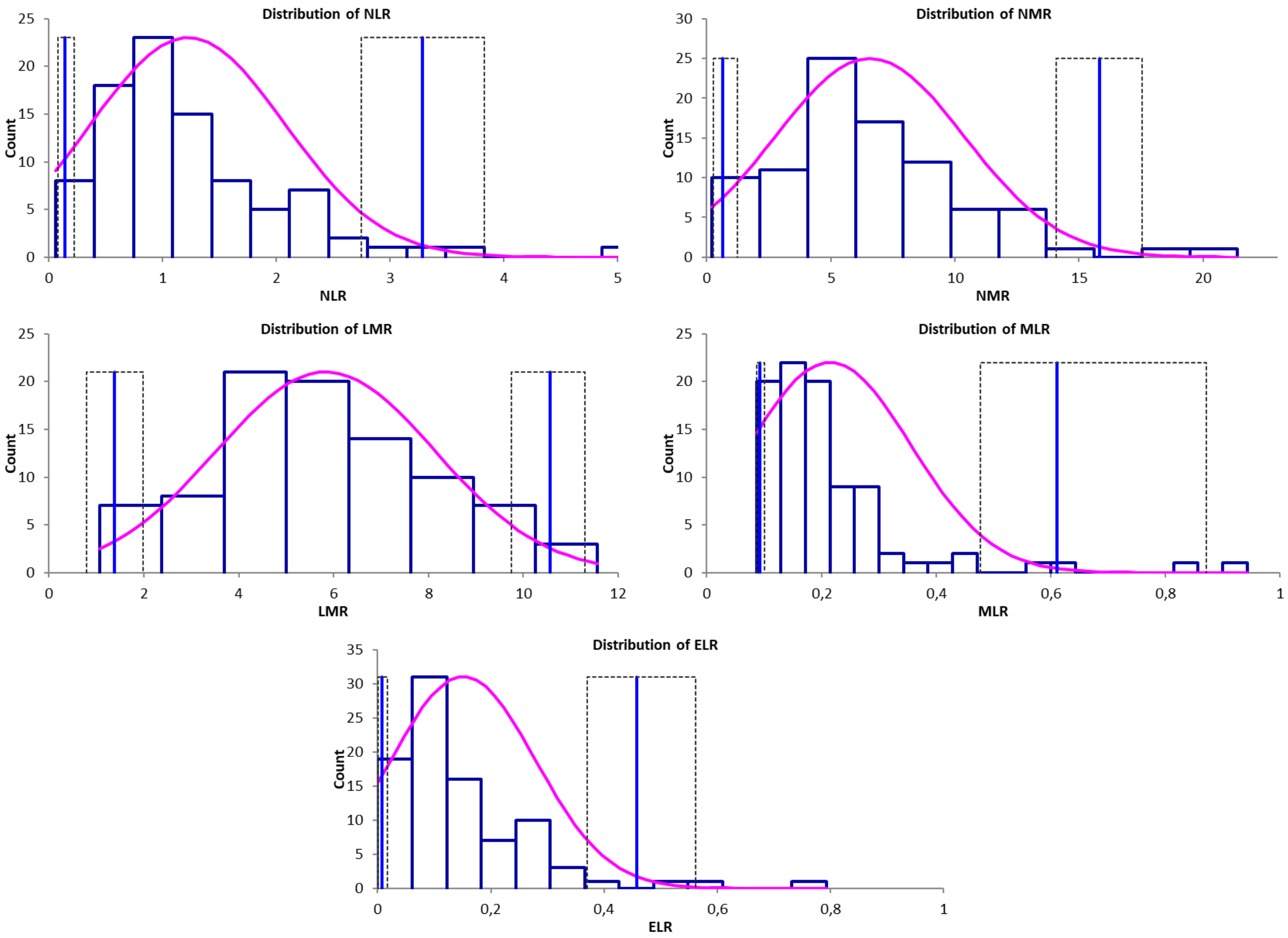

Supplementary Figure S1. Frequency distributions and reference intervals for NLR, NMR, LMR, MLR and ELR in healthy adult donkeys. The observed distribution is represented by the vertical black columns, while the red curve is the fitted distribution. Reference limits are drawn as vertical blue lines. Dotted bar surrounding those limits are the 90% confidence intervals.

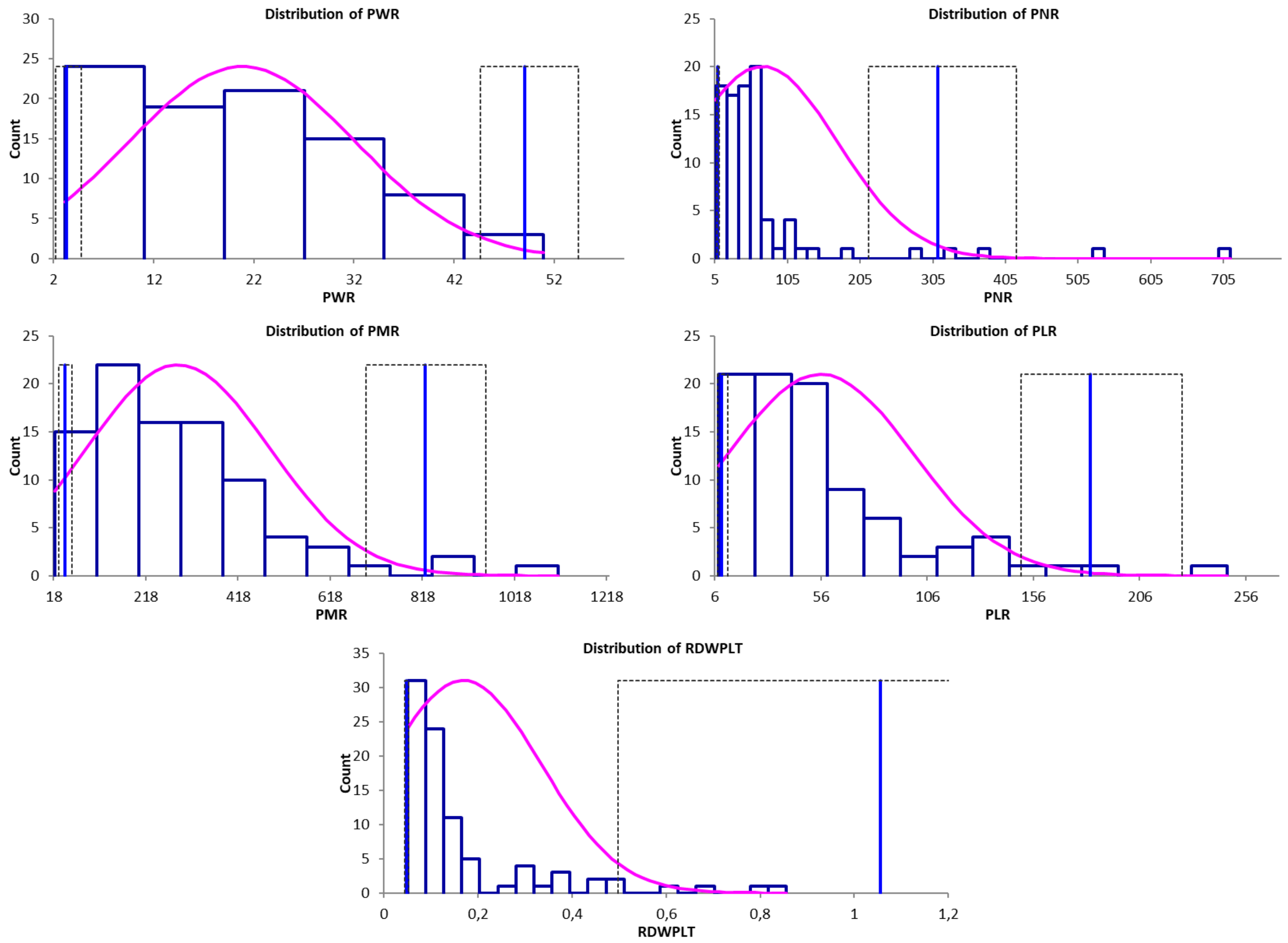

Supplementary Figure S2. Frequency distributions and reference intervals for PWR, PNR, PMR, PLR and RDWPLT in healthy adult donkeys. The observed distribution is represented by the vertical black columns, while the red curve is the fitted distribution. Reference limits are drawn as vertical blue lines. Dotted bar surrounding those limits are the 90% confidence intervals.

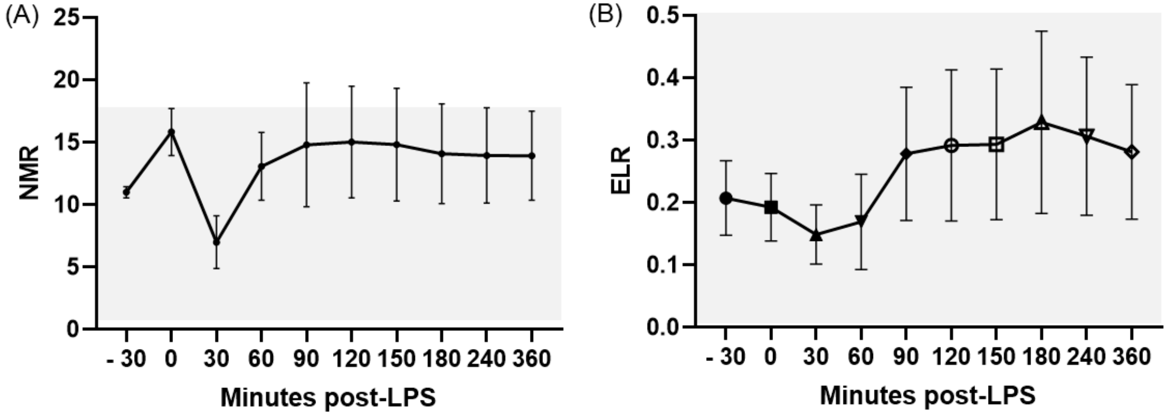

Supplementary Figure S3. NMR (A) and ELR (B) in donkeys after LPS infusion. Bar represents the standard error of the mean. The grey area represents the reference ranges for healthy donkeys. * $p < 0.05$ vs. -30 minutes (base-line). NMR, neutrophil to monocyte ratio; ELR, eosinophil to lymphocyte ratio.

Supplementary Table S1. Reference intervals for hematologic ratios in healthy donkeys (n = 90).

Ratio	LRL of RI	URL of RI	CI 90% of LRL	CI 90% of URL
NLR (Neutrophil to Lymphocyte)	0.1	3.6	0.1 – 0.2	2.6 – 5.2
NMR (Neutrophil to Monocyte)	0.3	16.8	0.2 – 0.6	12.7 – 21.4
LMR (Lymphocyte to Monocyte)	1.1	10.4	0.5 – 1.7	9.8 – 11.0
MLR (Monocyte to Lymphocyte)	0.1	0.8	0.1 – 0.1	0.4 – 0.9
ELR (Eosinophil to Lymphocyte)	0.0	0.6	0.0 – 0.0	0.3 – 0.8
PWR (Platelet to WBC)	3.4	47.8	3.1 – 6.0	37.6 – 51.0
PNR (Platelet to Neutrophil)	6.9	488.1	6.0 – 11.9	255.7 – 714.3
PLR (Platelet to Lymphocyte)	8.6	178.9	7.7 – 12.7	143.8 – 247.4
PMR (Platelet to Monocyte)	46.1	878.5	20.6 – 64.8	638.8 – 1112.5
RDWPLT (RDW to Platelet)	0.1	0.8	0.1 – 0.1	0.5 – 0.9

CI, confidence interval; ELR, eosinophil to lymphocyte ratio; LMR, lymphocyte to monocyte ratio; LRL, lower reference limit; MLR, monocyte to lymphocyte ratio; NLR, neutrophil to lymphocyte ratio; NMR, neutrophil to monocyte ratio; PLR, platelet to lymphocyte ratio; PMR, platelet to monocyte ratio; PNR, platelet to neutrophil ratio; PWR, platelet to WBC ratio; RDWPLT, red cell distribution width to platelet ratio; RI, reference interval; URL, upper reference limit.
